# Synthesis of novel C-doped g-C_3_N_4_ nanosheets coupled with CdIn_2_S_4_ for enhanced photocatalytic hydrogen evolution

**DOI:** 10.3762/bjnano.10.92

**Published:** 2019-04-18

**Authors:** Jingshuai Chen, Chang-Jie Mao, Helin Niu, Ji-Ming Song

**Affiliations:** 1 School of Chemistry and Chemical Engineering, Anhui University, Hefei 230601, P.R. China

**Keywords:** C-doped g-C_3_N_4_, CdIn_2_S_4_, composite materials, hydrogen generation, photocatalysis

## Abstract

Photocatalytic hydrogen generation from water splitting has become a favorable route for the utilization of solar energy. An effective strategy, the combination of C-doping with nanocomposite semiconductors, is presented in this work. C-doped g-C_3_N_4_ (CCN) was prepared by supramolecular self-assembly and subsequently a number of CdIn_2_S_4_/CCN composite photocatalysts were designed and fabricated though in situ decoration of CdIn_2_S_4_ crystals on the surface of CCN nanosheets via a hydrothermal method. This unique architecture was able to efficiently promote the transfer and separation of photon-generated charges, enhance light absorption, and significantly increase photocatalytic H_2_ production. Detailed characterization was performed to analyze the crystal structure, morphology, elementary composition, optical properties and catalytic mechanism. The CdIn_2_S_4_/CCN nanocomposites with optimal CdIn_2_S_4_ content exhibited a maximum H_2_ production rate of 2985 μmol h^−1^ g^−1^, almost 15 times more than that obtained using pure g-C_3_N_4_ (205 μmol h^−1^ g^−1^). In addition, the hybrid photocatalysts display good recycling stability under visible-light irradiation. This research may provide promising information for the preparation of more efficient multifunctional hybrid photocatalysts with excellent stability in fine chemical engineering.

## Introduction

The serious environmental concerns and increasing global energy demand have instigated growing awareness in the field of alternative energy generation over the past few decades. Photocatalysis technology based on semiconductor materials is a promising strategy for advancing the utilization of solar energy to the level of viable industrial production, such as organic synthesis [1–2], environmental governance [3–4], as well as fuel production [5–6].

Graphitic carbon nitride (g-C_3_N_4_), as a novel metal-free organic catalysts with visible-light response, has been extensively used in pollutant elimination, hydrogen production and photoreduction of CO_2_ because of its facile fabrication, superior physicochemical stability, appropriate energy band structure, and low cost [7–9]. Nevertheless, the photocatalytic activity of g-C_3_N_4_ is severely restricted by the inefficient separation of photogenerated electron–hole pairs and insufficient photon absorption. Up to now, a variety of strategies such as anion doping, novel metal deposition on surfaces and the design of heterojunctions/nanocomposites have been devoted to improving the transport and separation of electron–hole pairs [10–16]. Among them, C atom doping for N atoms in g-C_3_N_4_ is highly promising due to its π-rich nature, which can evidently improve the photocatalytic performance of g-C_3_N_4_ [17–21]. For instance, Huang et al. fabricated self-doped C-atom g-C_3_N_4_ via self-assembly, which exhibited highly efficient photocatalytic activity of H_2_ evolution under visible-light irradiation [22]. Moreover, the construction of a heterojunction composite is an effective approach to facilitate the separation of photogenerated holes and electrons. Yang et al. designed and constructed a 2D/2D nanocomposite photocatalyst through the in situ generation of ZnIn_2_S_4_ nanoleaf structures on the surface of g-C_3_N_4_ nanosheets by a facile one-step solvothermal method with surfactant, which exhibited distinct high-speed charge transfer nanochannels [5]. The as-prepared g-C_3_N_4_ nanosheet@ZnIn_2_S_4_ nanoleaf structure displays an enhanced photocatalytic activity for H_2_ production without the addition of a Pt co-catalyst.

As visible-light-active photocatalysts, ternary metal sulfide (e.g., ZnIn_2_S_4_ and CdIn_2_S_4_) have attracted great attention because of the suitable band edge and band gap, as well as tunable optical properties [23–28]. For example, CdIn_2_S_4_ has been reported in various photoredox catalysis, such as organic photosynthesis, CO_2_ photoreduction and H_2_ evolution [29–31]. Despite these advances, the photocatalytic performance of CdIn_2_S_4_ alone is barely satisfactory, mainly due to the low separation and migration efficiency of photogenerated charge carriers. The construction of a heterojunction by combination with semiconductor materials is expected to be a strategy to improve the separation of photogenerated charge carriers and further improve the performance of photocatalysts. Wang et al. synthesized In_2_S_3_–CdIn_2_S_4_ nanotubes with hierarchical heterostructure by a self-templated method, which exhibited efficient and stable photocatalytic activity of CO_2_ reduction under visible-light irradiation [32]. In addition, hierarchical CdIn_2_S_4_/graphene nano-heterostructures have been fabricated as efficient photocatalysts for solar H_2_ evolution [33].

In this study, synergy can be obtained by combining the two strategies, self-doped C-atom g-C_3_N_4_ (CCN) and hybridization with another semiconductor, to enhance photocatalytic activity. Accordingly, the two-step fabrication of CdIn_2_S_4_/CCN photocatalysts with different CdIn_2_S_4_ content is the target of this study. It is demonstrated that the CdIn_2_S_4_/CCN hybrid shows a superior H_2_ production activity without the addition of a Pt co-catalyst under visible-light irradiation.

## Results and Discussion

### Preparation and characterization of photocatalysts

**Figure 1:**
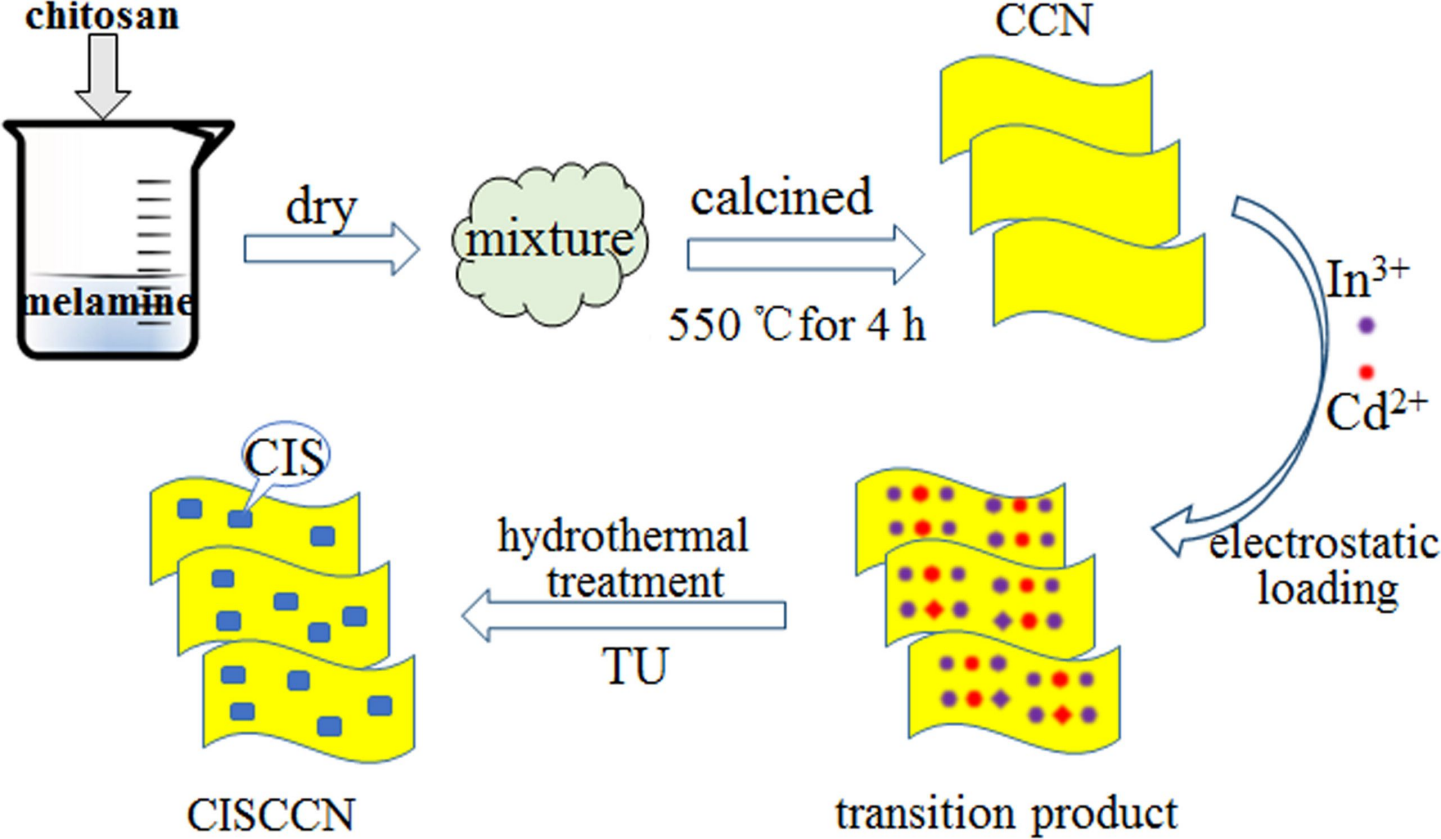
Schematic illustration of the fabrication procedure of CdIn_2_S_4_/CCN composite photocatalysts.

The preparation procedure of CdIn_2_S_4_/CCN photocatalysts is demonstrated in Figure 1. Firstly, carbon-bridged g-C_3_N_4_ (CCN) was prepared by a simple supramolecular self-assembly process using melamine and chitosan as precursors. Subsequently, the CCN was dispersed in an aqueous solution containing thiourea, Cd(NO_3_)_2_·4H_2_O and In(NO_3_)_3_·4.5H_2_O. As a result of the electrostatic attraction between cations and negatively charged CCN [34], Cd^2+^ and In^3+^ could easily load on to the surface of the CCN nanosheets. Thereafter, during hydrothermal treatment, the produced S^2−^ was able to react with absorbed Cd^2+^ and In^3+^ to generate CdIn_2_S_4_ on the CCN surface. As a consequence, these CdIn_2_S_4_/CCN binary composite photocatalysts were obtained.

**Figure 2:**
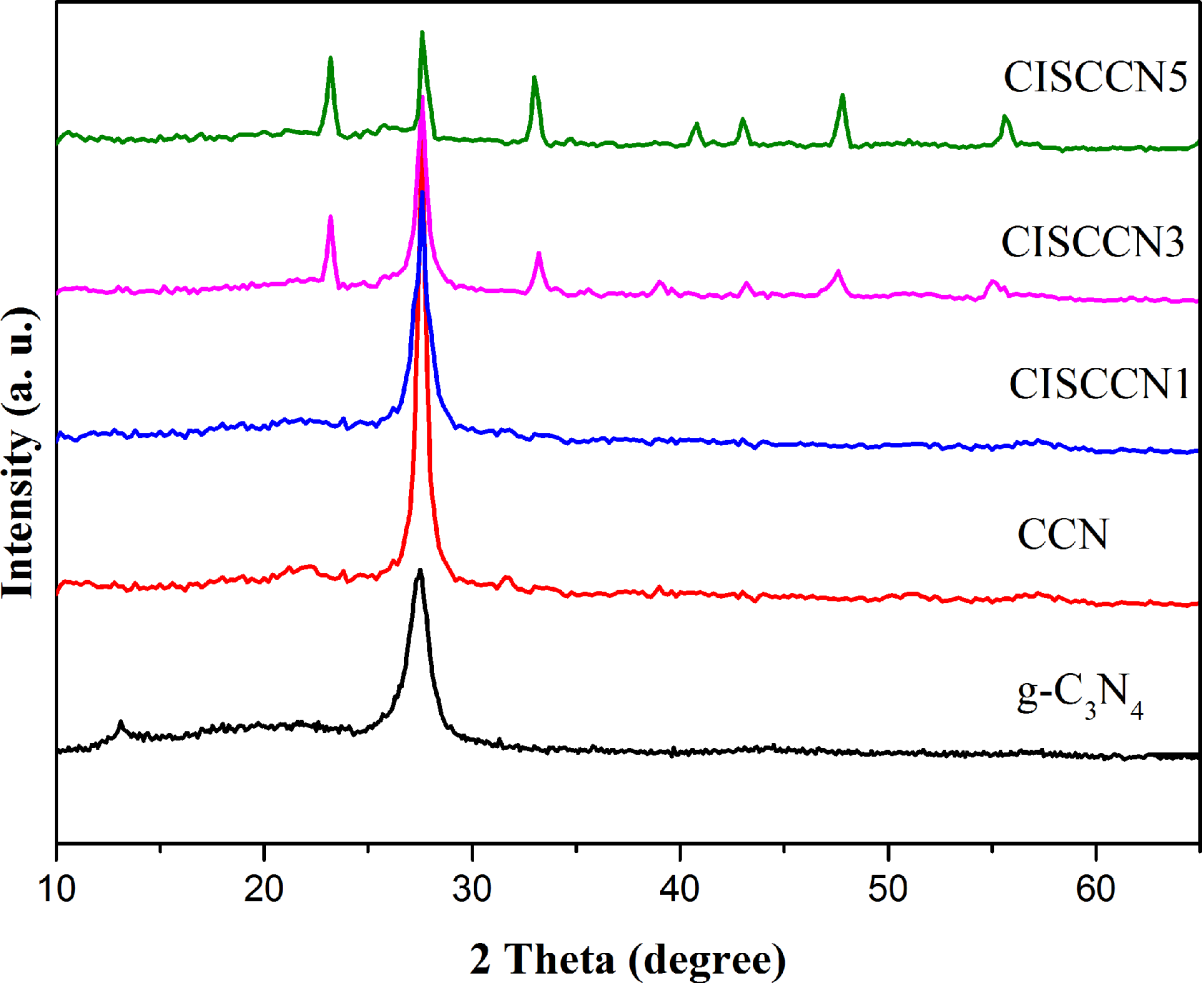
XRD patterns of the as-prepared samples.

The phase structure and purity of pure g-C_3_N_4_, CCN nanosheets, and the CdIn_2_S_4_/CCN (CISCCN) composite products were characterized by X-ray diffraction (XRD). As shown in Figure 2, the (100) peak located at 13.1° displayed in pure g-C_3_N_4_ is attributed to the in-planar stacking of tris-triazine units [35]. The characteristic graphite-like nanosheet structure of g-C_3_N_4_ is determined by the strong typical (002) peak at 27.6°, indicating that interplanar layers are packed along the direction perpendicular to the layer because of π–π interactions [36]. However, the peak at 13.1° for CCN is reduced compared to pure g-C_3_N_4_, which can be ascribed to the C element doping into the g-C_3_N_4_ crystal lattice [37]. The CISCCN samples exhibit similar characteristic XRD patterns as that of CCN due to the low content of CdIn_2_S_4_. With regard to the XRD patterns of the CISCCN3 and CISCCN5 composites, the peaks are in good agreement with (220), (311), (400), (422), (511), (440) and (533) crystallographic planes of the cubic CdIn_2_S_4_ (JCPDS no. 27-0060), revealing the presence of CdIn_2_S_4_ in the composites.

**Figure 3:**
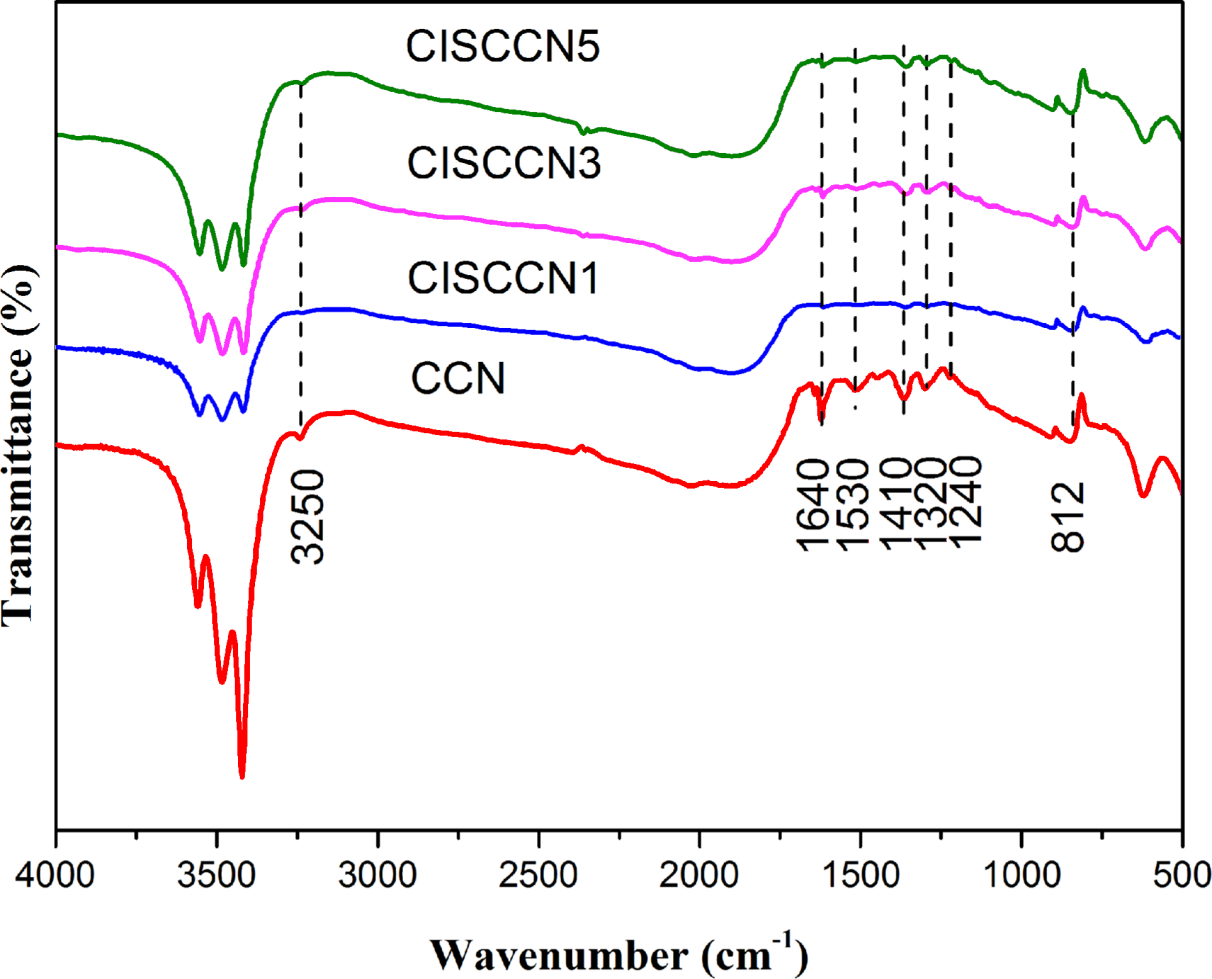
FTIR spectra of CCN, CISCCN1, CISCCN3 and CISCCN5 samples.

Figure 3 presents the Fourier transform infrared (FTIR) spectra of CCN and the CISCCN photocatalyts. In the case of CCN nanosheets, a series of peaks at 1240, 1320, 1410, 1530 and 1640 cm^−1^ between 1700 cm^−1^ and 1200 cm^−1^ are related to the typical stretching vibration of C–N and C=N in the CN heterocycles [38]. The characteristic peak of 812 cm^−1^ is due to the particular breathing mode for s-triazine (C_3_N_3_) units of g-C_3_N_4_ [7]. The FTIR absorption band at the region of >3200 cm^−1^ is ascribed to O–H of absorbed water and the stretching modes of uncondensed amine groups [4]. Obviously, after the combination of CdIn_2_S_4_ and CCN, the resulting CISCCN nanocomposites possess similar FTIR spectra as that of the CCN sample.

**Figure 4:**
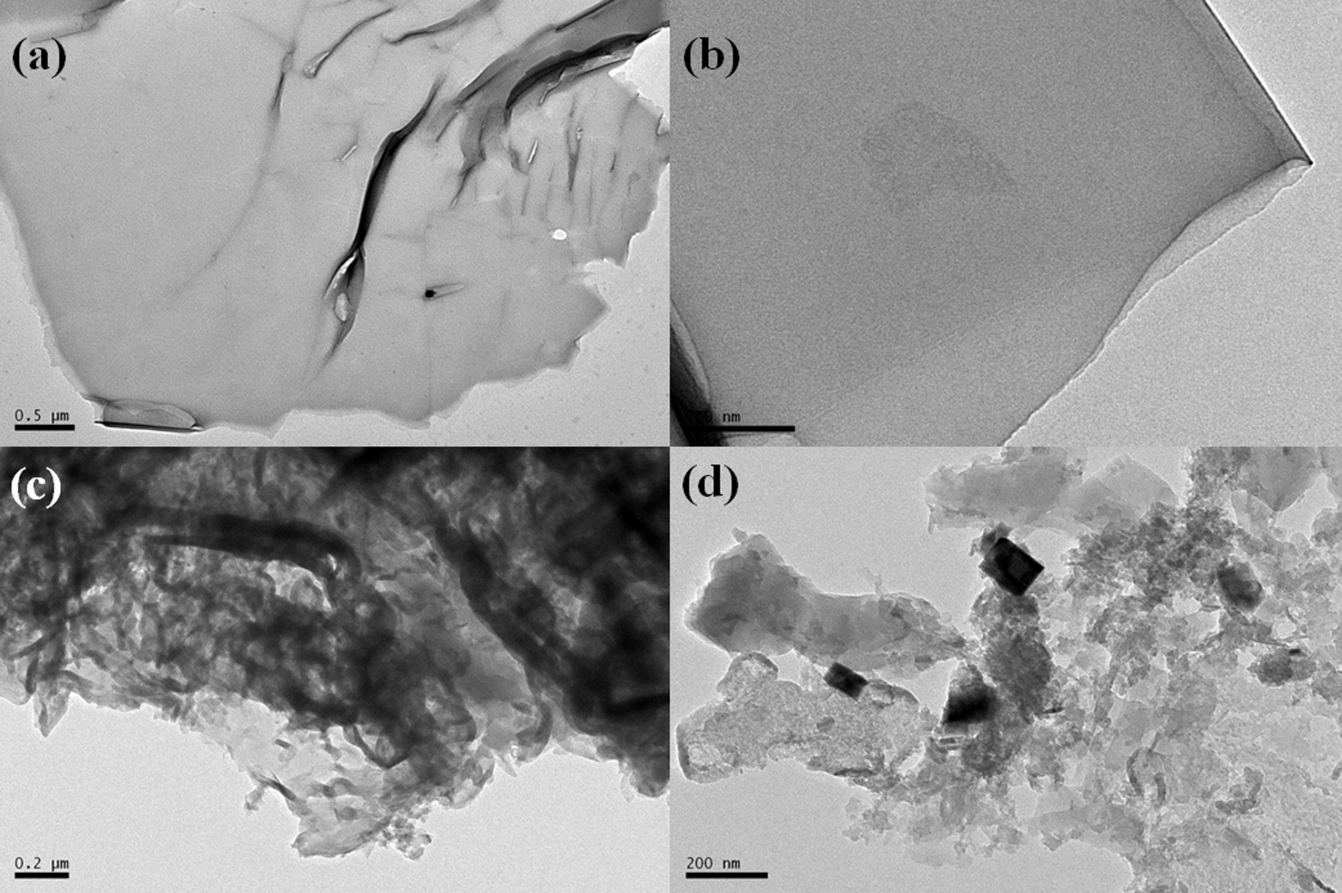
TEM images of (a,b) CCN and (c, d) CISCCN3 samples.

The microstructure and morphology of the as-fabricated CCN and CISCCN3 samples were characterized by transmission electron microscopy (TEM) images. As can be seen in Figure 4a,b, the CCN sample displays a representative two-dimensional layered structure that is ultrathin. After the addition of CdIn_2_S_4_ to form the heterojunction with CCN, the CdIn_2_S_4_ crystals are attached to the CCN surface, resulting in close contact (Figure 4c,d).

**Figure 5:**
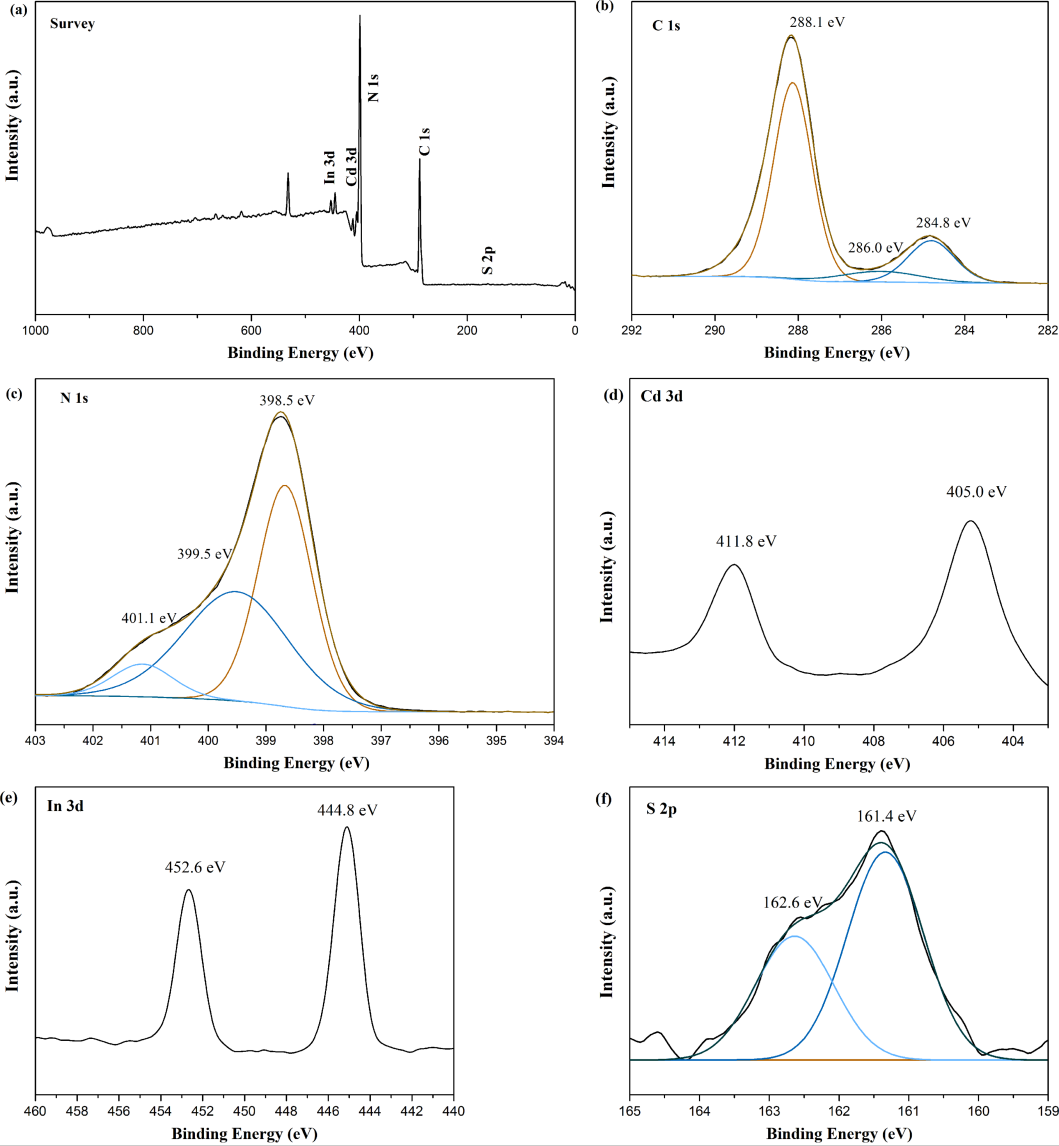
XPS spectra of CISCCN3 composites: (a) survey spectrum, (b) C 1s, (c) N 1s, (d) Cd 3d, (e) In 3d and (f) S 2p.

X-ray photoelectron spectroscopy (XPS) was utilized to study the surface elemental composition of the representative composite photocatalyst CISCCN3. The survey XPS spectrum of Figure 5a shows that the CISCCN3 composite is mostly composed of C, N, Cd, In, and S, also verifying the coexistence of CCN nanosheets and CdIn_2_S_4_ nanocrystals in the composite. The C 1s spectrum in Figure 5b shows some new peaks with binding energies at 284.8 eV, 286.0 eV and 288.1 eV. According to the reported literature, the peak located at 284.8 eV belongs to the carbon reference, and the peaks at 286.2 eV and 288.1 eV are attributed to the C–N=C bonds and C–(N)_3_ groups of CCN [30]. The N 1s spectrum (Figure 5c) can also be deconvoluted into three new peaks located at 398.5 eV, 399.5 eV, and 401.1 eV, which can be ascribed to the sp^2^-hybridized N atoms in the C=N–C (aromatic rings), N–(C)_3_ (tertiary nitrogen) and C–N–H (amino functional groups with H atoms), respectively [30]. As shown in Figure 5d, the Cd 3d spectrum reveals two peaks located at 405.6 eV and 412.3 eV, which correspond to the peaks of Cd 3d_5/2_ and 3d_3/2_, respectively [39]. The photoelectron peak for In 3d (Figure 5e) exhibits two peaks, corresponding to In 3d_5/2_ and In 3d_3/2_ [40]. Moreover, the S 2p peak splitting of 161.4 eV and 162.6 eV, a split energy with 1.2 eV, represents the S^2−^ in the nanocomposite sample [28]. Consequently, from the XPS results, it can be concluded that the CdIn_2_S_4_ has been successfully deposited on CCN nanosheets through the heterojunction formation, promoting the transfer of photo-induced charge between these two materials. No other peaks appeared in the XPS spectrum of CISCCN, inferring that impurities have not been introduced during the composite preparation process.

**Figure 6:**
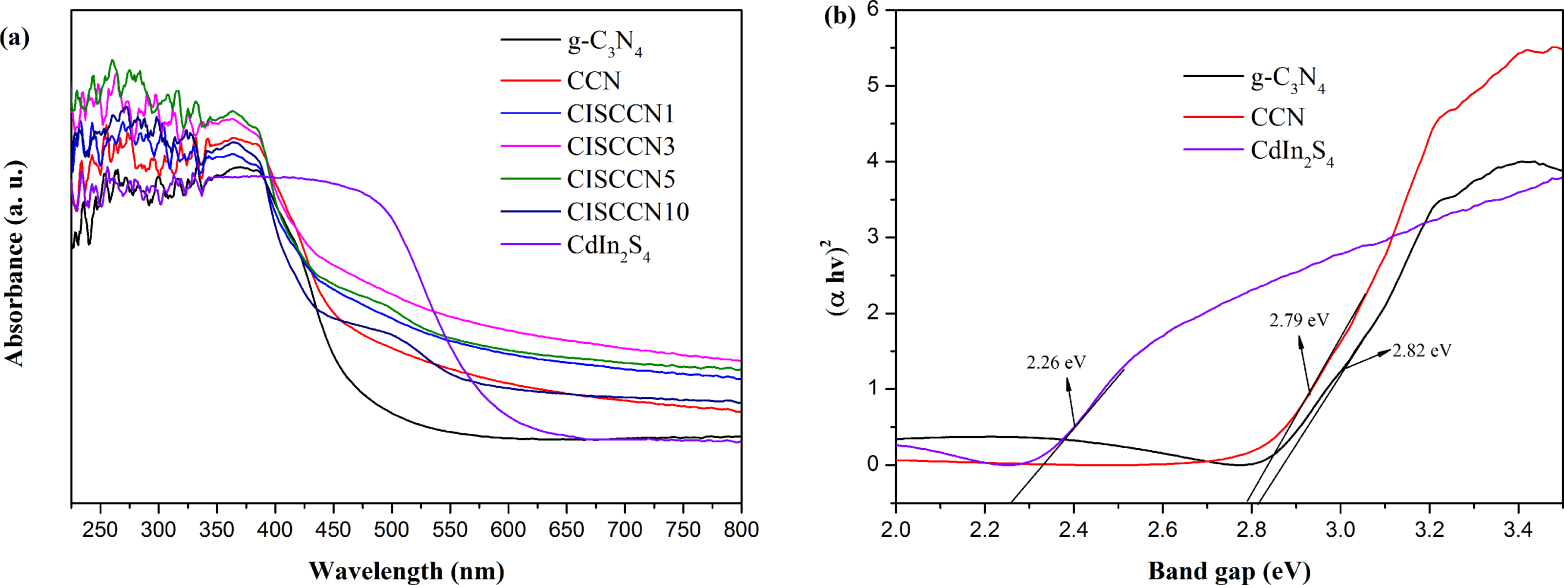
(a) UV–vis spectra of g-C_3_N_4_, CCN, CdIn_2_S_4_, and CISCCN composites. (b) The plot of (α*h*ν)^2^ vs photon energy (*h*ν) for g-C_3_N_4_, CCN and CdIn_2_S_4_.

UV–vis diffuse reflectance spectroscopy (DRS) was used to investigate the optical absorption properties of g-C_3_N_4_, CCN and the series of CISCCN composites. As shown in Figure 6a, g-C_3_N_4_ exhibits an absorption edge at approximately 426 nm. For the CCN sample, however, the light absorption background strengthens in the longer wavelength region (λ > 450 nm), which can be obviously noticed. The light absorption in the prepared CCN sample is greatly improved, owing to the formation of large delocalized π bonds. Compared with pure CCN, all the CISCNN composites exhibit wider absorption in the visible-light range. This phenomenon may be ascribed to the introduction of CdIn_2_S_4_ with slightly narrower bandgaps and the band alignment of the two materials. Furthermore, the introduction of CdIn_2_S_4_ into the matrix of CCN has an important impact on the optical absorption properties of CISCCN nanocomposites. It can be clearly observed that the light absorbance decreases in the visible light range upon addition of CdIn_2_S_4_ content in CISCCN nanocomposites. The band gap (*E*_g_) of g-C_3_N_4_, CCN and CdIn_2_S_4_ can be estimated by plotting (α*h*ν)^2^ as a function of the photon energy (Figure 6b), with α being the absorption coefficient, *h* being Planck’s constant, and v being the frequency. The *E*_g_ of g-C_3_N_4_, CCN and CdIn_2_S_4_ are estimated as 2.82 eV, 2.79 eV and 2.26 eV, respectively. Besides, the position of the valence band is determined by the following equation: *E*_VB_ = *X* − *E*_c_ − 0.5*E*_g_, where *E*_c_ is the energy of free electrons on the hydrogen scale (about 4.5 eV), *X* is the electronegativity of the semiconductor, and *E*_g_ is the band gap energy of the semiconductor. The edge of the valence band (VB) and conduction band (CB) of g-C_3_N_4_, CCN and CdIn_2_S_4_ are summarized in Table 1.

**Table 1:** The calculated values of the band gap energy (*E*_g_), valence band (VB) and conduction band (CB).

Samples	*E*_g_ (eV)	VB (eV)	CB (eV)

g-C_3_N_4_	2.82	1.54	−1.28
CCN	2.79	1.53	−1.26
CdIn_2_S_4_	2.26	1.47	−0.79

**Figure 7:**
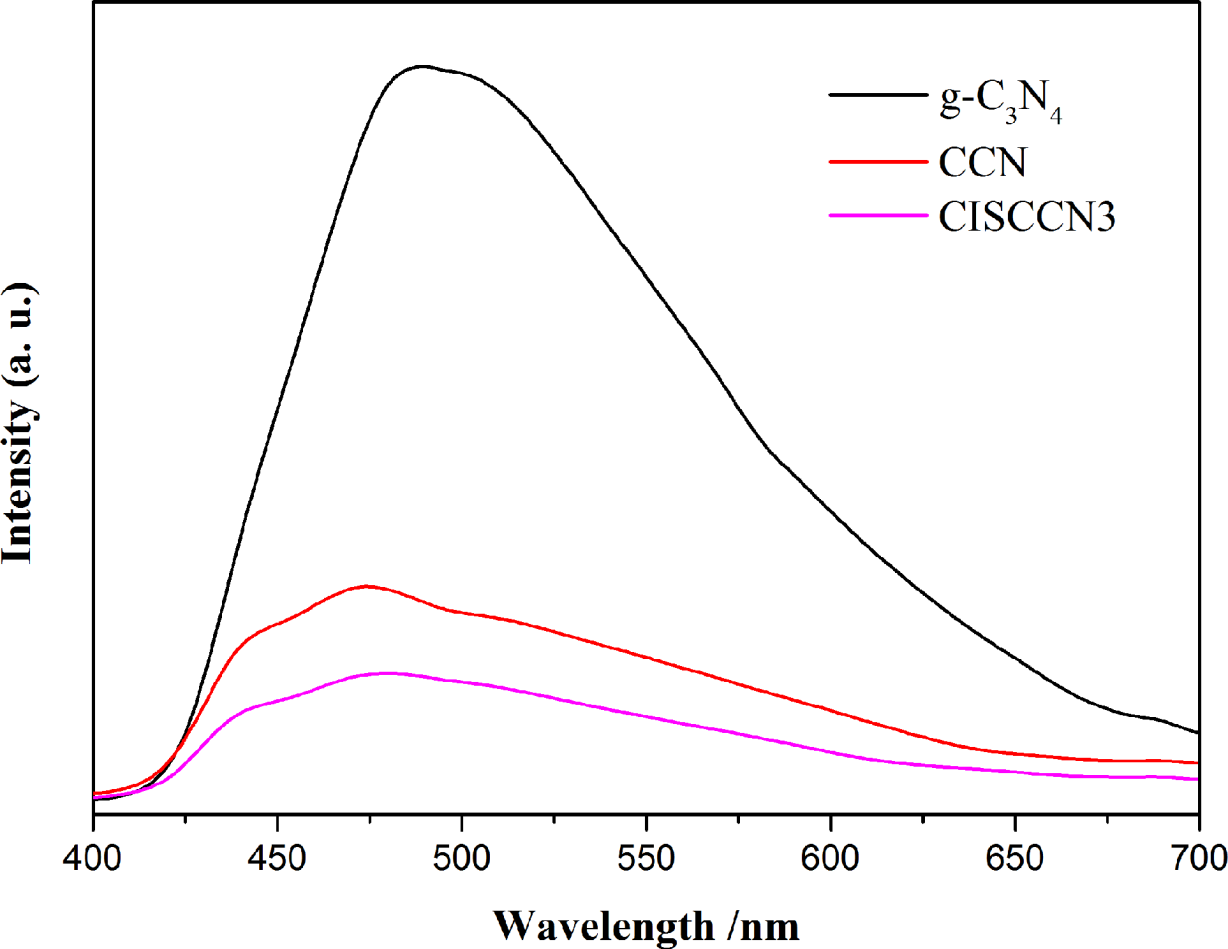
Photoluminescence spectra of g-C_3_N_4_, CCN and CISCCN3 samples.

The separation–recombination rate of photo-induced charge carriers of these as-fabricated samples has been studied via room temperature photoluminescence (PL) characterization. Figure 7 displays the PL emission spectra of g-C_3_N_4_, CCN and CISCCN3 hybrid photocatalysts at the excitation wavelength of 410 nm. It is observed that the pure CCN shows one strong emission peak located at about 482 nm, resulting from band gap recombination of photogenerated carries of g-C_3_N_4_. Compared with pristine g-C_3_N_4_, the emission intensity peaks of the CCN samples decreases evidently, indicating that the separation rate of photogenerated electron–hole pairs over CISCCN3 composites is remarkably facilitated. In addition, a further decrease in the PL emission intensity in the CISCCN3 hybrid photocatalyst can be observed. This result demonstrates that the construction of a heterogeneous interface can improve the separation and transfer of photogenerated charges apparently, leading to the enhancement of photocatalytic activity of the hybrid photocatalyst.

### Photocatalytic performance

**Figure 8:**
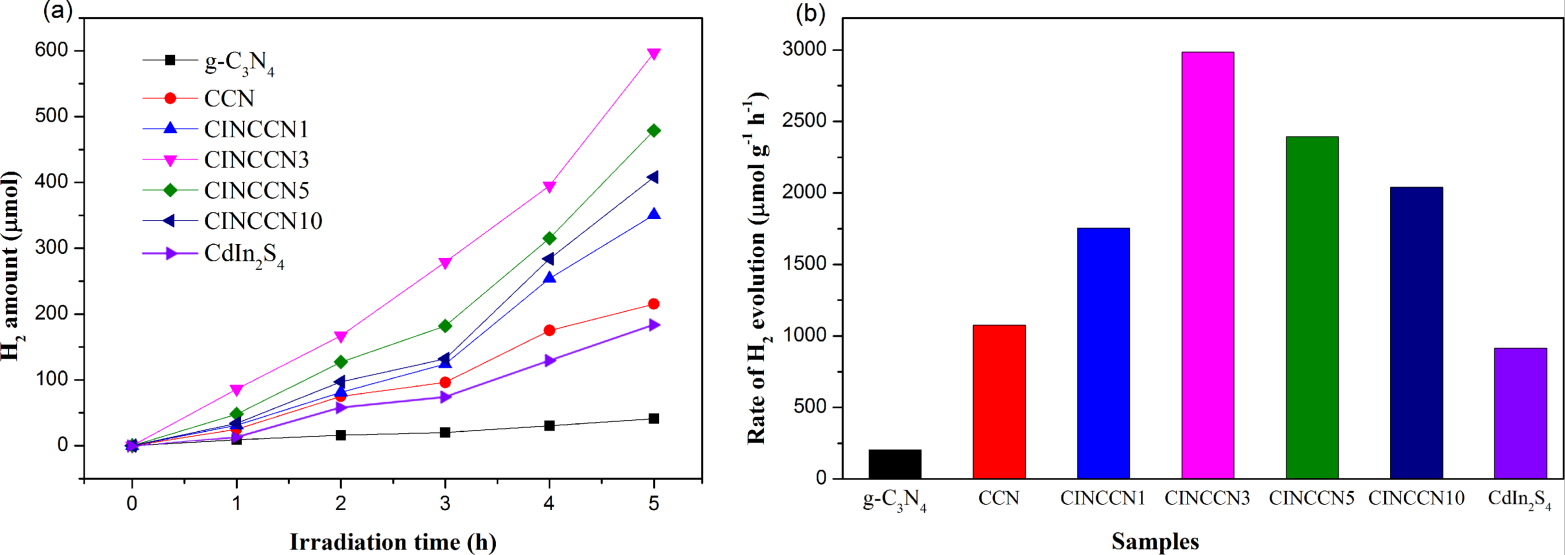
(a) Time-dependent visible-light-induced photocatalytic H_2_ formation over different samples without incorporation of Pt as the co-catalyst. (b) H_2_ formation rates of different samples.

Time-dependent visible-light-induced photocatalytic H_2_ formation over different samples has been measured in the presence of methanol (sacrificial electron donor) without the use of the additive Pt co-catalyst. As shown in Figure 8, the pristine g-C_3_N_4_ presents a negligible H_2_ generation rate, but after introduction of the self-doped C by a simple supramolecular self-assembly method, the CCN nanosheets display a higher H_2_ generation rate due to the presence of the large, delocalized π bonds. Moreover, the pure CdIn_2_S_4_ sample also shows a low H_2_ generation rate. Regarding the CISCCN binary hybrid composites, the photocatalytic H_2_ generation is evidently increased. The H_2_ generation rate reaches a maximum of 2985 μmol h^−1^ g^−1^ over CISCCN3. A further increase of CdIn_2_S_4_ loading results in a decline in the photocatalytic performance for H_2_ evolution. It is revealed that excessive loading of CdIn_2_S_4_ may have an adverse effect on the photo-redox catalytic reaction owing to the blocking effect that impedes the light absorption of CCN [41]. This result corresponds well with the results from UV–vis spectra.

**Figure 9:**
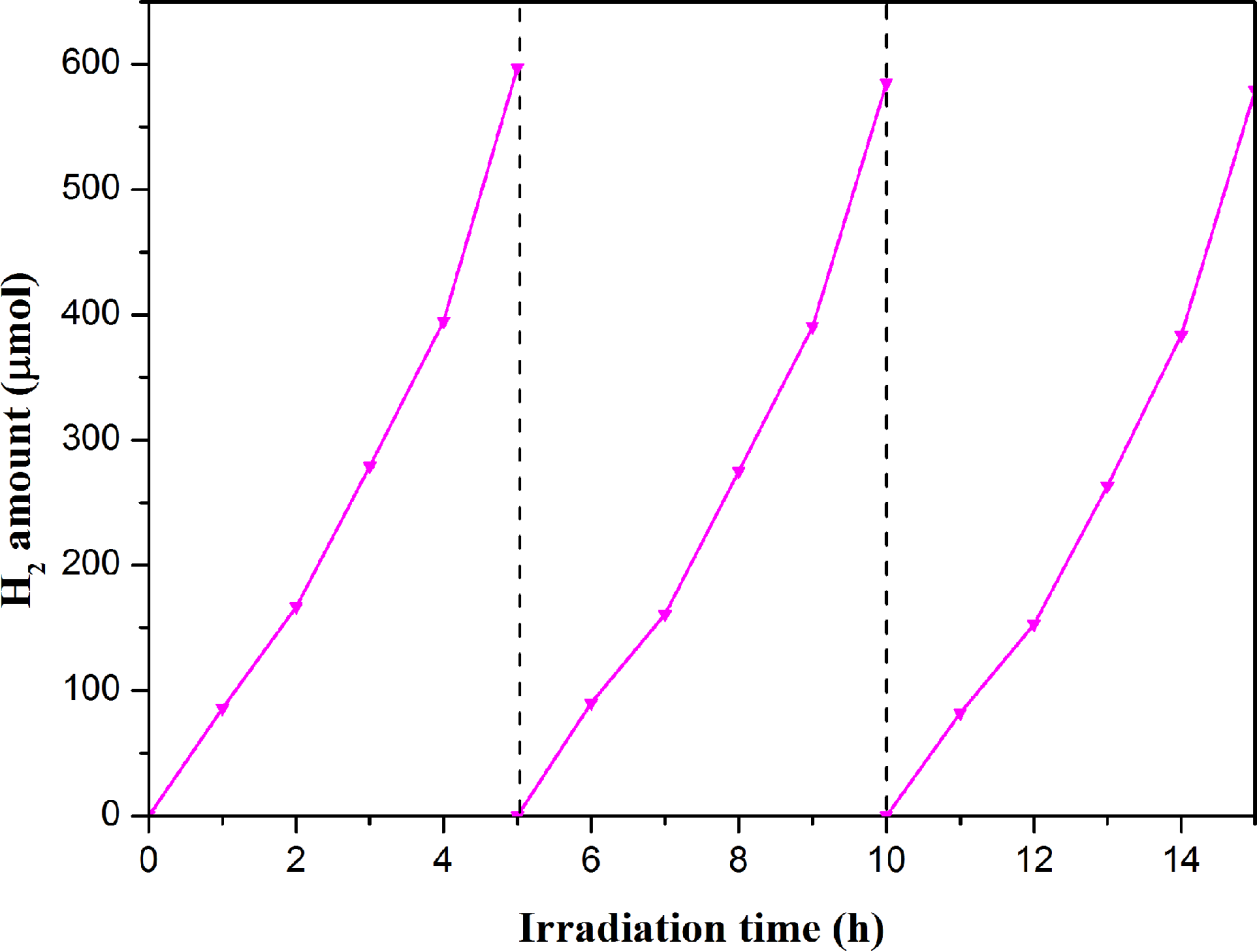
Cycling study of photocatalytic H_2_ formation over CISCCN3.

In view of practical applications, in addition to the photocatalytic H_2_ generation rate, the stability and durability are also significant factors to test the feasibility for application of the photocatalysts. The stability and durability of the most efficient CdIn_2_S_4_/CCN (CISCCN) sample, the CISCCN3 photocatalyst, is further investigated in recycling photocatalytic experiments. As illustrated in Figure 9, the photocatalytic performance of the CISCCN3 sample presents no obvious loss after three cycles after an accumulative 15 h under the same experimental conditions.

**Figure 10:**
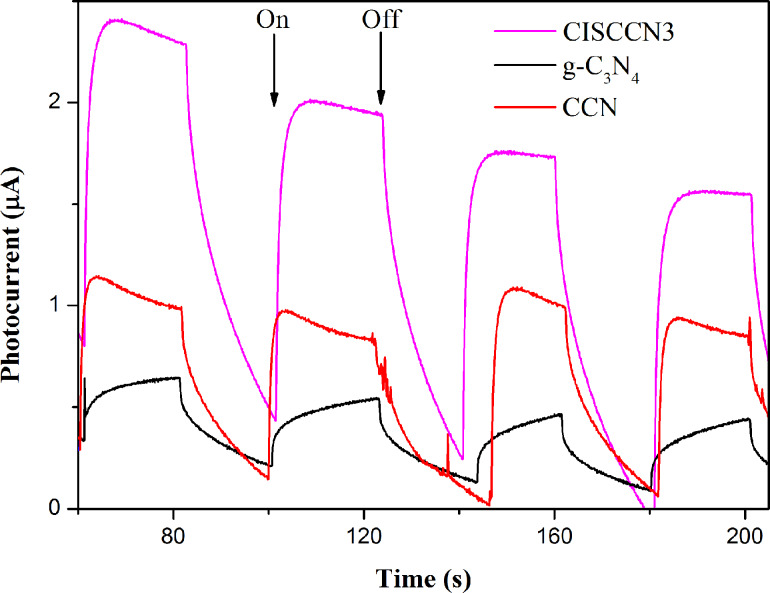
Transient photocurrent response of g-C_3_N_4_, CCN and CISCCN3 under visible-light irradiation.

Based on these results, it is hypothesized that high-efficiency photocatalytic H_2_ generation activity is the consequence of C-doping and formation of a heterogeneous interface, which is an important factor in facilitating the separation of photogenerated electron–hole pairs. In order to further confirm this, a transient photocurrent response measurement is employed to evaluate the charge migration and separation efficiency of the as-obtained photocatalysts. Transient photocurrents of g-C_3_N_4_, CCN and CISCCN3 are studied during the on–off cycles with intermittent exposure of visible-light excitation (Figure 10). Evidently, the as-prepared CCN and CISCCN3 samples display a distinct increase of photocurrent intensity in comparison with single CCN, demonstrating that C-doping as well as the close heterogeneous junction formed between CdIn_2_S_4_ and CCN can facilitate the separation of photogenerated electrons and holes, which ultimately endows the CISCCN3 composites with the enhanced photocatalytic performance. This photocurrent result corroborates the results from the UV–vis DRS and PL experiments, as well as the photocatalytic performance.

### Photocatalytic mechanism

**Figure 11:**
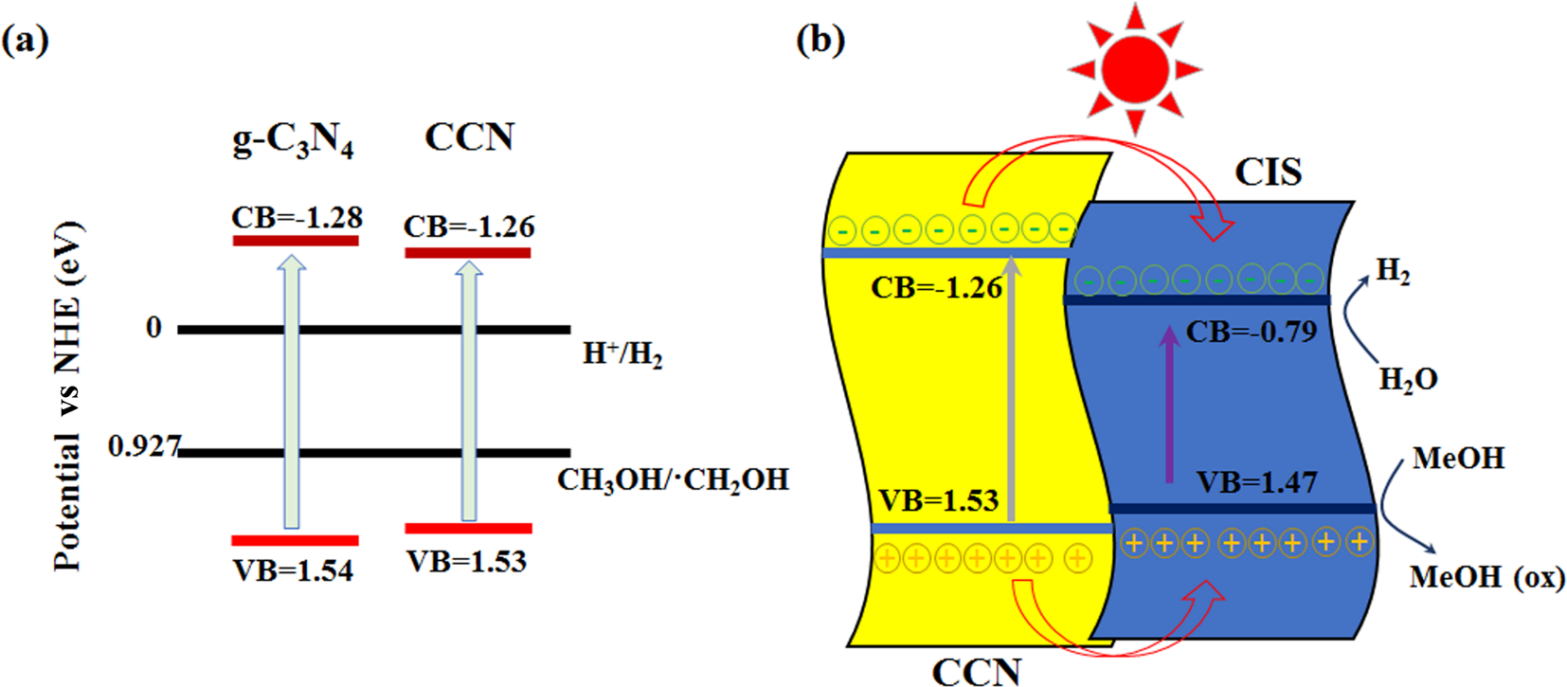
(a) Band structure alignments for g-C_3_N_4_ and CCN. (b) Proposed mechanism of photocatalytic H_2_ generation for CdIn_2_S_4_/CCN composites under visible-light irradiation (λ ≥ 420 nm).

Figure 11a displays the specific position of the conduction and valence bands of the g-C_3_N_4_ and CCN samples. CCN has a narrower band gap than the original g-C_3_N_4_ because of the C-doping, allowing for more visible light to be harvested. Furthermore, C-doping can increase the π-electron availability and reduce the hydrogen absorption energy [42]. As presented in Figure 11b, a possible mechanism of charge transfer for H_2_ formation has also been proposed over CISCCN heterostructured photocatalysts under visible-light irradiation. The type-I binary heterojunction interfaces can be formed because of the proper VB and CB positions for CCN and CIS, improving the charge transfer/separation efficiency. Upon visible-light irradiation, both CCN nanosheets and CIS can absorb photons to produce massive electron–hole pairs, then electrons in the VB of the semiconductors are able to be excited to the CB, leaving holes in the VB. Because the CB edge potential of CIS (−0.79 eV) is more positive than that of CCN (−1.26 eV), the photo-induced electrons in the CB of CCN can readily transfer to the CB of CIS and then reduce the hydrogen ions to produce H_2_ molecules. At the same time, the photogenerated holes in the VB of CCN can flow into that of CIS, which are quickly consumed by methanol as the sacrificial electron donor. Accordingly, the bulk recombination rate of the photogenerated carriers is effectively inhibited, further improving the photocatalytic activity for H_2_ generation.

## Conclusion

In summary, CdIn_2_S_4_/C-doped g-C_3_N_4_ composite photocatalysts have been prepared for photocatalytic hydrogen evolution under visible-light illumination. This approach not only results in a material with a narrower bandgap but also in the efficient charge transfer and separation, realizing the synergistic effect of C-doping and composite nanostructures. It was found that CISCCN nanocomposites show significant photocatalytic activity for H_2_ generation, especially the CISCCN3 sample. In addition, the CISCCN3 sample was found to be highly stabile during the cycled photocatalytic reaction. This study offers new insights for the application of g-C_3_N_4_ based composite photocatalyts with enhanced visible-light absorption and highly efficient charge migration and separation.

## Experimental

### Sample preparation

Self-doped carbon/g-C_3_N_4_ structures were prepared according to the literature [32]. In detail, 1 g of melamine powder was first dispersed in 300 mL of deionized water with continuous stirring. Subsequently, 0.01 g of chitosan was dissolved in this solution. The resultant solution was stirred for 4 h at room temperature, and dried at 80 °C. Finally, the mixture was ground into powder and calcined at 550 °C for 4 h with a heating rate of 5 °C min^−1^ under air atmosphere. After cooling down to room temperature naturally, the obtained g-C_3_N_4_ product (CCN) was collected. For comparison, pure g-C_3_N_4_ was also prepared following the same steps without chitosan, which was labeled as g-C_3_N_4_.

The CdIn_2_S_4_/CCN composites were synthesized via a hydrothermal method. Typically, an appropriate amount of Cd(NO_3_)_2_·4H_2_O, In(NO_3_)_3_·4.5H_2_O and thiourea (TU) were added into 60 mL of deionized water, followed by 15 min of ultrasonication. Meanwhile, 0.5 g of as-obtained CCN powder was dispersed in the above solution by ultrasound for 1 h. The suspension was transferred into a 100 mL Teflon-lined stainless steel autoclave, sealed and maintained at 180 °C for 12 h, then cooled naturally and washed with ethanol and distilled water. The product was dried at 60 °C for 10 h. The as-prepared CdIn_2_S_4_/CCN samples with 1, 3, 5 and 10 wt % CdIn_2_S_4_ were denoted as CISCCN1, CISCCN3, CISCCN5 and CISCCN10, respectively. Pure CdIn_2_S_4_ (CIS) was also synthesized by the same hydrothermal method but without adding CCN.

### Characterization

The as-prepared products were characterized by transmission electron microscopy (TEM) (JEM-2100, JEOL, Japan), Fourier-transform infrared spectroscopy (FTIR, NEXUS-870, Nicolet Instrument Co. USA), X-ray diffraction (XRD, XD-3, Purkinje General, China, Cu Kα radiation), X-ray photoelectron spectroscopy (XPS, Escalab 250Xi, America), UV–vis diffuse reflectance spectroscopy (DRS, Hitachi U-4100) at a wavelength range of 200–800 nm, and fluorescence photospectroscopy (Hitachi F-4500).

### Photocurrent measurements

The photocurrent response of the photocatalyts were measured using a standard three-electrode electrochemical station (CHI 660D, Chenhua Instrument Co., Ltd, Shanghai, China). In this system, the Ag/AgCl electrode was chosen as the reference electrode and a Pt wire was used as the counter electrode, respectively. The electrolyte was a 1 M Na_2_SO_4_ aqueous solution. A glassy carbon electrode containing the as-prepared sample served as the working electrode.

### Photocatalytic H_2_ production reaction

In this work, the activity of the photocatalyst was tested in a closed circulation reactor system for in situ photocatalytic H_2_ production (CEL-SPH2N, AuLight, Beijing) at 6 °C and −0.1 MPa. Before the reaction, 40 mg of the photocatalyst was dispersed into 40 mL of aqueous methanol solution (CH_3_OH/H_2_O = 1:4 v/v). A 300 W Xe lamp (CEL-HXF300, AuLight, Beijing) equipped with a 420 nm optical filter (to cut off ultraviolet light) was placed on top as the light source. The system was degassed before irradiation and the evolution of hydrogen was detected by inline gas chromatography (CEL-GC7920, TCD, 5A molecular sieve column and N_2_ as carrier gas).
